# From problematic soil to engineered fill: Transforming granite residual soil with clay improvement

**DOI:** 10.1371/journal.pone.0344432

**Published:** 2026-03-06

**Authors:** Fang Dang, Hong Guo, Ya Wang, Wenyang Li, Hong Jiang, Jiangtao Fu

**Affiliations:** 1 School of Civil Engineering and Architecture, Shaanxi University of Technology, Hanzhong, China; 2 Student Research Society of Human Settlements, Shaanxi University of Technology, Hanzhong, China; 3 Research Center of Geotechnical Environment and Geological Hazards Control in Qinling-Daba Mountains, Shaanxi University of Technology, Hanzhong, China; Graphic Era Deemed to be University, INDIA

## Abstract

To investigate the enhancement effect of clay on the mechanical properties of granite residual soil in the Qinba Mountains of China, this study systematically examined the influence of clay content on the liquid and plasticity index, compaction behavior, compressibility, cohesion and internal friction angle of the soil. The analysis used a range of laboratory tests. These include tests to measure liquid and plastic limit determinations. The analysis also used tests for compaction and compression and tests that examine direct shear. Observations of structures at small scale provided additional data. The findings show that particles of clay fill spaces in soil. This filling increases how compact the soil is. The filling also improves capacity to hold water. Hydroxyl groups that the clay introduces allow this improvement. The adsorption of water molecules by the clay also supports this effect. When the clay content increases, the plasticity index shows increase and the maximum moisture content shows increase in the granite residual soil. The clay content that reaches 30% produces the maximum dry density that shows the peak value in the soil, and this represents increase of approximately 21.4% when results compare to the soil without improvement. The compression coefficient decreases to the minimum level at the same clay content, and this reflects reduction of 75%. The cohesion of the soil mass shows increase that relates to increasing clay content in a linear pattern. The internal friction angle shows gradual decrease with the clay content increase, and this angle tends to stabilize when the clay content exceeds 30%. These findings indicate that clay provides effective improvement material for the engineering properties that the granite residual soil contains. The approach that uses clay offers reliable reference for applications that involve roadbed filling.

## 1 Introduction

Granite forms a main component of the crust in continents. This rock shows distribution across southern China, with area of exposure of 86 × 104 km^2^, which represents approximately 9% of total land area in China [1–4]. Over time periods of billions of years, processes of weathering that continue over long periods transform granite into a thick layer of soil that shows complete weathering and remains at the surface, which is commonly referred to as residual soil from granite. In comparison with other soils that form through settling of material [5], residual soil derived from granite is characterized by poor grain-size distribution, low compaction density, low cohesion, high susceptibility to softening, and pronounced disintegration tendency upon wetting or mechanical disturbance [6–7]. These properties relating to the ground that are not favorable increase risk of damage in a significant manner and also increase risk of failure in engineering of foundations, construction of subgrade, and stability of slope. In recent years, challenges in engineering that include settling of subgrade, instability of slope, landslides, and flows of debris that are attributed to instability that is inherent in residual soil from granite show prevalence that is increasing.

Researchers examine methods that provide improvement to features of granite residual soil. Methods using physical approaches show effects. The use of materials such as geosynthetic forms and fibers in soil allows increase in strength by increasing friction within the material [8–9]. Zeity et al. [10] examine the use of slaked lime and pineapple fiber for providing improvement to granite residual soil. Results indicate that additives increase the strength measure for soil under compression without constraint by 31% and 26% at curing times of zero days and seven days. Ferreira et al. [11] examine behavior of high-density polyethylene (HDPE) unidirectional geogrids that occur in granite residual soil under single loading and multiple loading in cycles. Findings reveal that total movement of HDPE under loading in cycles increases with higher initial load and with higher load range. This movement decreases with increase in loading rate and with increase in soil density. Ferreira et al. [12] also observe that strength at the interface between residual soil and geosynthetic materials decreases with increase in soil moisture level. This strength increases significantly with greater soil density. Chemical methods for providing stability also receive study. These include the use of materials such as cement, lime, and fly ash. Su et al. [13] demonstrate that cement addition provides improvement to compaction features, to capacity for bearing load, to strength under compression without constraint, and to strength under dynamic loading of granite residual soil. Tang et al. [14] examine a soil treatment that combines kaolin, lime, and cement. This treatment provides increase to water resistance of the soil surface. It also increases the capacity of the soil for holding water. Liu et al. [15] found through extensive triaxial and disintegration tests that adding 6% cement effectively improved the shear strength and disintegration resistance of granite residual soil, making it suitable for practical engineering applications. However, physical approaches provide limited improvement to soil mass cohesion. Although chemical stabilization techniques are highly effective, the production of cement and lime emits substantial amounts of carbon dioxide, posing significant environmental concerns.

Identifying soil treatment that shows more favorable effects for the environment and provides effectiveness is particularly important. Clay contains large amounts of minerals that form in layers such as kaolinite, illite, and montmorillonite [16], and this material provides advantages that include low cost and availability in local areas. Multiple studies demonstrate that clay produces significant improvements in properties relating to mechanical performance of soils. Niu et al. [17] used tests examining compaction and findings indicate that including 8% and 4% clay produced notable effects on behavior during compaction and capacity for bearing in weathered granite residual soil from the Luliang Mountains, and this addition provided conditions that allow the soil to reach optimal performance for bearing loads. Gao et al. [18] present findings that show incorporating 6% red clay produced effective increases in cohesion and strength during shearing of granite residual soil, while including 18% produced significant improvements in the internal friction angle of the soil mass. Similarly, Sun et al. [19] demonstrate that kaolin improves and increases the mechanical properties of granite residual soil using characteristics that provide cementation that this material contains.

Studies in the past show important developments for improving features relating to the mechanical function of granite residual soil, but work examining clay that comes from the Qinba Mountains to improve the mechanical features of granite residual soil from this area appears limited.In view of the above research deficiencies, this paper adopts different indoor tests such as compaction tests and direct shear tests, as well as microscopic tests, to systematically study the improvement effect of natural clay on the mechanical properties of granite residual soil, providing an experimental basis for the green and environmentally friendly improvement scheme of “using local materials”. The findings provide valuable insights for improving the compaction performance as well as the cohesion of granite residual soil through a green and environmentally friendly improvement approach of “utilizing local materials”.

## 2 Experimental materials

### 2.1 Ethical statement

This study conducted sampling in a non-privately-owned area that is not governed by environmental or other cultural protection regulations. A review of local regulations confirmed that no special permits are required for sampling activities at this location. The study did not involve the use of, or any interference with, the life cycles or habitats of endangered or protected species, and no disturbance to the sampling environment occurred.

### 2.2 Physical properties of soil samples

**Fig 1:**
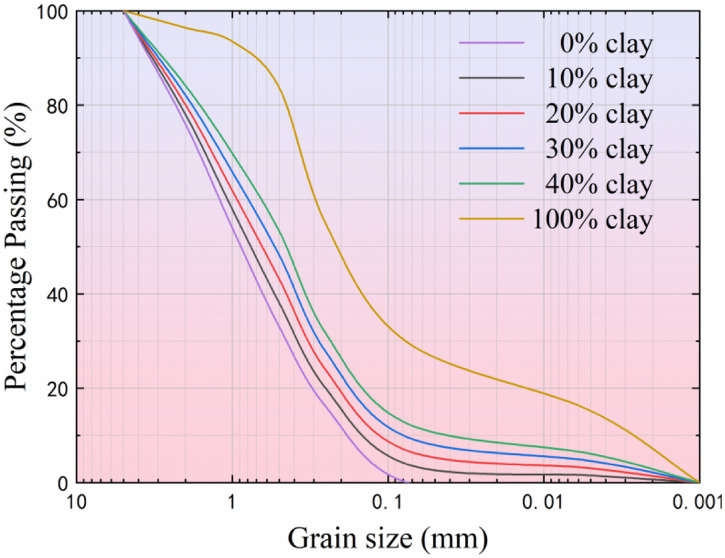
Particle size distribution of granite residual soil and clay.

**Fig 2:**
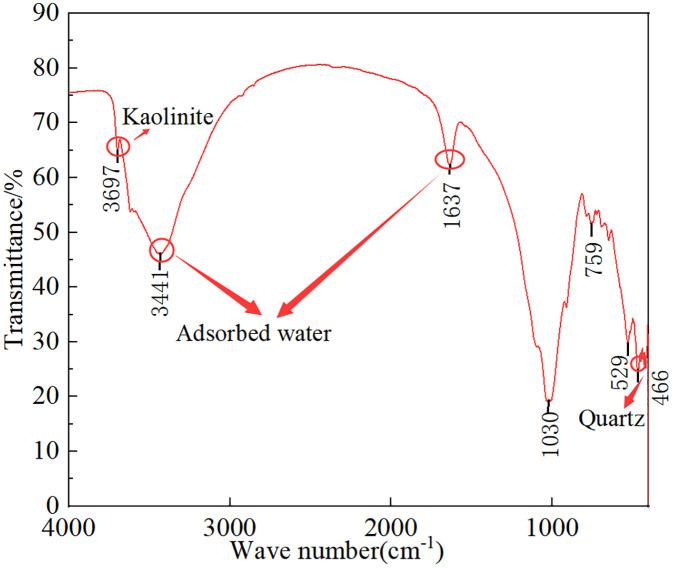
Fourier transform infrared spectroscopy of granite residual soil.

The granite residual soil was collected from a road slope in Tianming Town, Chenggu County, Hanzhong City, while the clay was sourced from a road slope in Hedongdian Town, Mian County, Hanzhong City. The clay is characterized as a slope-deposited gravelly clay. Both the granite residual soil and clay were naturally air-dried, crushed, and sieved through a 2 mm sieve. Their physical properties were measured and presented in Table 1, with the particle size distribution shown in Fig 1. The Fourier transform infrared spectrum of the granite residual soil shown in Fig 2 indicates that the main mineral components of the granite residual soil in this area are kaolinite and quartz, and it contains a large amount of adsorbed water. This is the internal cause that leads to the instability of its engineering mechanics properties and the easy occurrence of potential hazards such as slope instability. This mineralogical and hydrous composition constitutes the intrinsic factor contributing to the instability of its engineering mechanical properties and the susceptibility to potential geotechnical hazards, such as slope failure.

**Table 1:** Basic physical indexes of granite residual soil and clay.

Soil sample	Plastic limit (%)	Liquid limit (%)	Maximum dry density (g/cm^3^)	Optimum moisture content (%)	Plasticity index
**Clay**	15	35	1.67	15.1	20
**Granite residual soil**	20.5	27.1	1.54	6.51	6.6

### 2.3 Test method

#### (1) Sample preparation.

**Fig 3:**
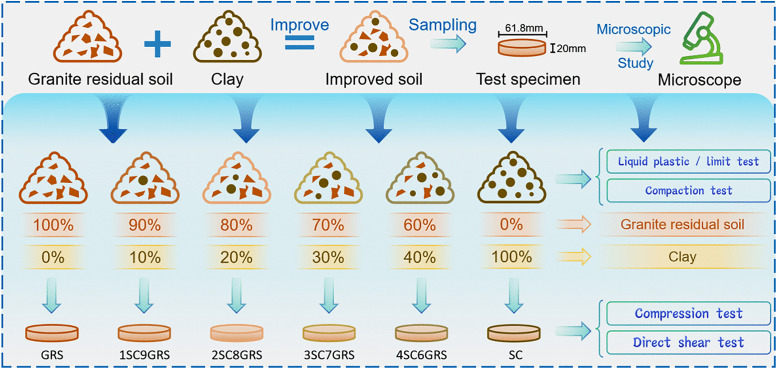
Sample preparation and testing process diagram.

As shown in Fig 3, this experiment employed the control variable method. Air-dried and sieved granite residual soil and clay were selected and mixed in proportions of clay mass to total mass of 0%, 10%, 20%, 30%, 40%, and 100%. The specimens used in the compression and direct shear tests had a diameter of 61.8 mm and a height of 20 mm. Prior to sample preparation, the required amount of water and the mass of soil were calculated based on the optimum dry density and the optimum moisture content. Water was added by spraying, followed by thorough mixing, sealing, and storage for 6 hours to ensure complete and uniform distribution of moisture within the soil samples. Additionally, to ensure homogeneous blending of the two soil types, the dry soils were first mixed using a mechanical mixer for 5 minutes, after which water was added and mixing continued for an additional 10 minutes until the mixture was uniform and free of aggregates. After compaction molding, the specimens were stored in a constant-temperature chamber at 20°C for 24 hours to allow moisture equilibration throughout the sample, followed by drying in an oven at 30°C. For the compaction tests and liquid and plastic limit tests, the prepared wet soil mixtures were sealed in plastic bags and maintained in a constant-temperature environment at 20°C to achieve moisture equilibrium before testing.

#### (2) Test type.

**Fig 4:**
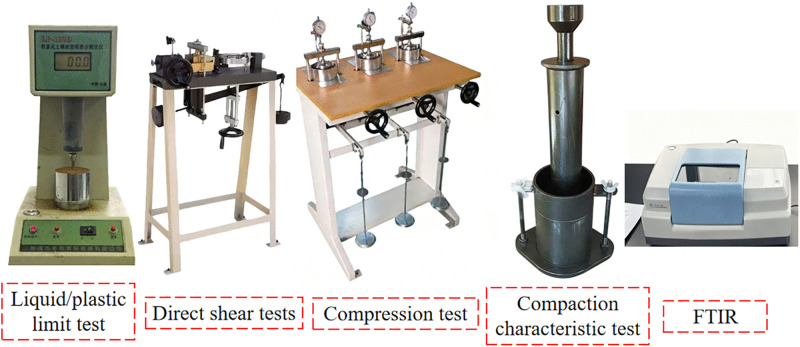
Test instrument.

According to the “Standard for Soil Test Methods” (GB/T 50123−2019), the liquid/plastic limit test,compaction characteristic test,compression and direct shear tests were conducted using the instrument shown in Fig 4.

Liquid-plastic limit test: A combined liquid-plastic limit apparatus was employed to determine the Atterberg limits. For each soil mixture, five specimens with varying moisture contents were prepared. After placing each specimen into the soil cup, the penetration depth of the standardized cone was measured. By systematically adjusting the water content and conducting repeated measurements, the relationship curves between moisture content and cone penetration depth were established. These curves were subsequently used to determine the liquid limit and plastic limit, respectively.

Compaction test: The compaction test is conducted using the light compaction method. The sample was placed in three layers into the compaction cylinder, and each layer was compacted with a 2.5 kg hammer in 25 strikes. After the test is completed, the wet density and moisture content of the sample are measured, the dry density is calculated, the compaction curves of each ratio are drawn, and the maximum dry density and the optimal moisture content are determined. Each set of proportions is repeated three times, and the average of the results is taken to ensure reliability.

Direct shear test: Use a strain-controlled direct shear apparatus. Direct shear tests were conducted under four different vertical pressures (100, 200, 300, and 400 kPa), and the test process was carried out at a shear rate of 0.8 mm/min until failure. Record the shear displacement and shear stress, draw the shear stress-displacement curve, and determine the cohesion c and internal friction Angle φ of each ratio under different normal stresses.

Compression test: The compression test was performed using a standard consolidation apparatus. The test was conducted under incremental loading conditions with applied stress levels of 12.5, 25, 50, 100, 200, 400, and 800 kPa. Each load increment was maintained until deformation reached a stable state. The compressive deformation at each stress level was recorded, and the void ratio and compression coefficient were calculated accordingly. Subsequently, the compression curve was plotted based on the obtained data.

Microscopic experiment: TESCAN-CLARA-LMH type scanning electron microscope was used to test the soil under scanning electron microscope. Granite residual soil and clay particles were extracted and examined under an optical microscope to observe their particle shapes. However, only particles with relatively clear contours were observed due to limitations of the microscope’s magnification.

Fourier Transform Infrared Spectroscopy (FTIR): The improved soil samples were air-dried, ground, and passed through a 200-mesh sieve. A total of 2 mg of soil particles was combined with 200 mg of potassium bromide (KBr) powder. The mixture was then finely ground in an agate mortar until a uniform consistency was achieved. Subsequently, the resulting blend was pressed into a transparent thin sheet using a tablet press at a pressure of 10 MPa. The spectrometer (Thermo Fisher Scientific Nicolet iS20, As shown in Fig 4) was activated, and the prepared soil tablets were placed in the sample chamber. The scanning parameters were set to a range of 4000–400 cm^-1^, with a resolution of 4 cm^-1^ and the number of scans configured to 32. Following the initiation of testing on the machine, the infrared absorption spectrum was obtained.

## 3 Results

This section may be divided by subheadings. It should provide a concise and precise description of the experimental results, their interpretation, as well as the experimental conclusions that can be drawn.

### 3.1 Liquid/Plastic limit test

**Fig 5:**
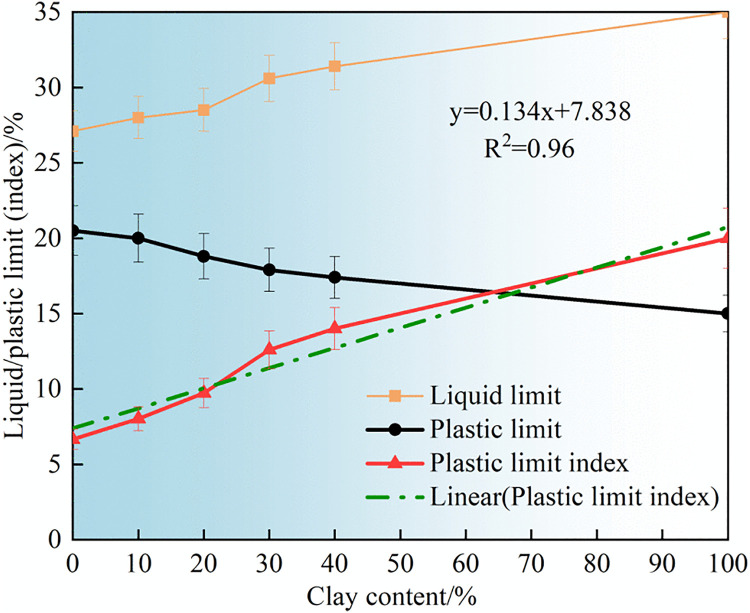
Changes in the liquid plastic limit of improved soil.

As shown in Fig 5, increasing clay content leads to a rise in the liquid limit and a decrease in the plastic limit of the soil. Notably, the growth rate of the liquid limit is relatively slow, whereas the plastic limit decreases more rapidly. The underlying mechanism lies in the fact that the liquid and plastic limits of soil are primarily governed by the combined influence of pore water and clay content within the matrix of coarse particles. However, the clay content in the residual soil of granite is low. The addition of clay can adsorb more water, thereby gradually increasing the water-holding capacity of the soil, and thus the fluid limit of the soil gradually grows. Meanwhile, as the clay fills the pores of the coarse particles, a lubricating water film is formed at the contact points between the soil particles, weakening the friction between the particles. Therefore, the plastic limit of the soil body rapidly decreases. The two changes jointly led to a significant increase in the plasticity index of the soil mass. Consequently, the plasticity index increases from an initial value of 6.66 to 20 as the clay content increases. This indicates that the addition of clay can significantly improve the water-holding capacity of granite residual soil.

Furthermore, it can be seen from the variation of plasticity index that it basically increases linearly with the content of clay (equation (1)). The inspiration for practical engineering is that local materials can be used to conveniently improve the water holding capacity of residual granite soil by adjusting the amount of clay added.


PI=ax+b (1)

Where x is the clay content, a= 0.134 and b=7.838 is the linear coefficients.

### 3.2 Analysis of compaction test data for improved soil

**Fig 6:**
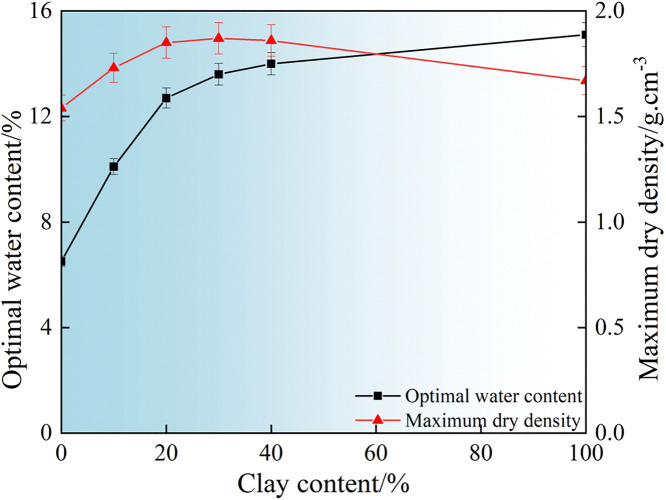
Improved soil compaction performance parameters.

Compaction tests on improved soil with different clay ratios revealed the optimal moisture content and maximum dry density curves, as shown in Fig 6. With increasing clay content, the optimal moisture content of the soil exhibits a gradual upward trend, rising from 6.51% to 15.1%, representing an approximate increase of 132%. The maximum dry density of the soil follows a pattern of initial increase followed by a decline, reaching its peak value of 1.87 g/cm^-3^ when the clay content is 30%. This phenomenon is mainly related to the rearrangement of soil particles and the interaction with pore water during the compaction process. When the clay content is low, clay particles can effectively fill the pores between the coarse-grained frameworks, improve the particle gradation, and make the soil more likely to reach a compact state. Therefore, during the test period, the maximum dry density of the soil mass increased with the increase of clay content. However, when the clay content is too high, fine particles gradually completely wrap around coarse particles, and the lubricating effect between particles is enhanced, making the particles more likely to slide during the compaction process, and the maximum dry density of the soil decreases instead. The increase in the optimal moisture content is related to the strong water absorption capacity of the clay particles themselves. The strong water absorption capacity makes the soil require more water to encapsulate the particles to exert its effect. When the content exceeds 20%, the soil’s water-holding capacity tends to be saturated. At this point, further addition of clay will weaken the water-holding capacity. Therefore, it is manifested as the growth rate of the optimal moisture content of the soil slows down as the clay content increases. This rule reflects the comprehensive influence of fine particle content on the interaction forces among particles, moisture and soil particles in the soil during the process from 0% to 100% clay content.

Based on a comprehensive analysis of the above research results, when the clay content is 30%. The maximum dry density of the “mixture” is the greatest. In filling engineering, it is recommended that the ratio of granite residual soil to clay be 7:3 to achieve the best compaction effect. Of course, 8:2 and 6:4 can also achieve ideal maximum dry densities. In actual filling projects, the mixing ratio can be flexibly selected based on the amount of these two types of soil materials.

### 3.3 Compression test

**Fig 7:**
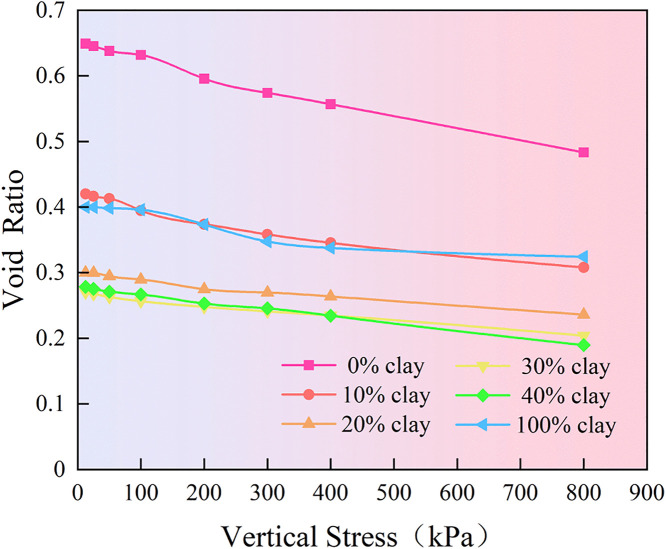
Verticle stress – void ratio curve.

**Fig 8:**
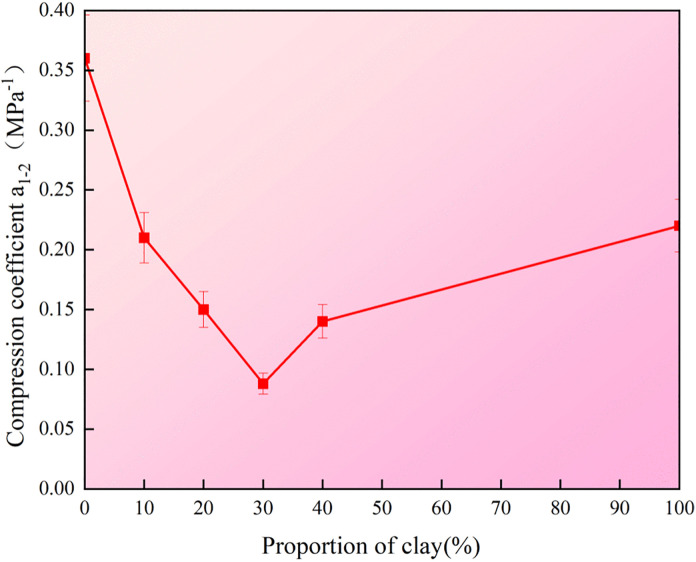
Proportion of clay – compression coefficient curve.

Furthermore, compression tests were conducted with the optimal moisture content and 95% compaction degree. The verticle stress – void ratio curve and compression coefficient of the mixture under different clay dosages are presented in Figs 7 and 8, respectively. The results indicate that the compressibility of granite residual soil varies systematically with increasing clay content. Overall, the void ratio of all samples decreases as vertical pressure increases. When the clay content is 0%, the soil exhibits the highest initial void ratio (0.65), and this ratio decreases more significantly with increasing vertical pressure. When the clay content is 30% and 40%, the initial pore ratio of the sample is the smallest, and the decrease in the pore ratio of the sample with the increase of vertical pressure is relatively gentle. As can be seen from Fig 8, the compression coefficient (the ratio of the change in porosity and vertical pressure difference at 100kPa and 200kPa) of the sample shows a trend of first sharply decreasing and then slowly increasing with the variation of clay content. When the clay content is 0%, the compression coefficient of the soil is 0.36MPa^-1^. When the content increases to 30%, the compressibility decreases to its minimum value (0.09 MPa^-1^), at which point the soil is classified as low-compressibility soil, making it favorable for engineering applications. After the clay content continues to increase to 100%, the compression coefficient of the soil will gradually increase to 0.22MPa^-1^. The reason for this phenomenon lies in that at a lower dosage, clay can effectively fill the large pores between soil particles, making the pore structure of the soil more compact. Therefore, it is manifested as a decrease in the pore ratio and a weakened compressive deformation capacity. However, at higher clay contents, the increased proportion of fine particles makes the soil more susceptible to particle slippage and reorganization under vertical loading, leading to a slower decrease in void ratio and an increase in the compression coefficient.

The above research results indicate that when the clay content reaches 30%, the initial porosity of the soil mass is minimized, and the compression coefficient remains below 0.1 MPa^-1^. This meets the standard of soil with low compressibility, indicating an ideal improvement effect. It should be noted that the prerequisite for this conclusion is that the compaction degree of the soil should reach 95%. In engineering applications, the compaction degree of the mixed soil used for filling should not be lower than this standard.

### 3.4 Analysis of direct shear test data for improved soil

**Fig 9:**
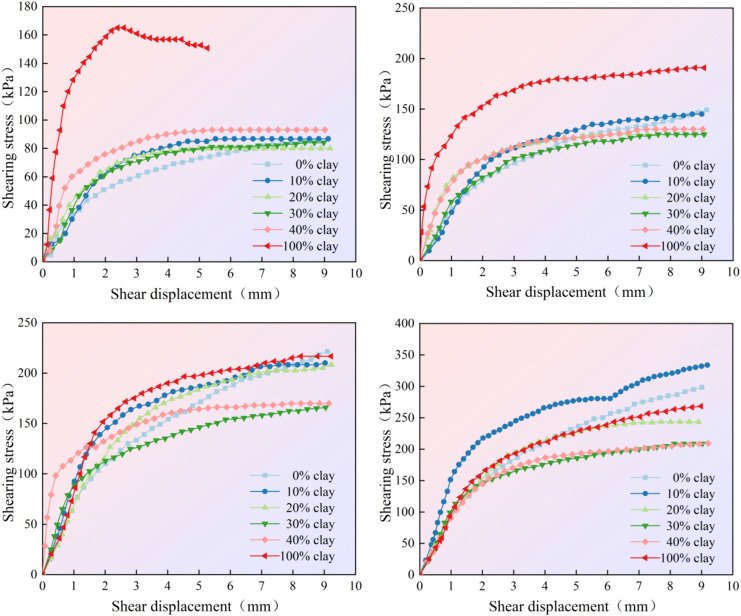
Shear displacement and shear stress curves under different vertical pressures.

The relationship between shear stress and shear displacement of clay-improved soil under vertical pressures of 100 kPa, 200 kPa, 300 kPa, and 400 kPa, as well as varying clay contents, was determined through direct shear tests, as displayed in Fig 9.

**Fig 10:**
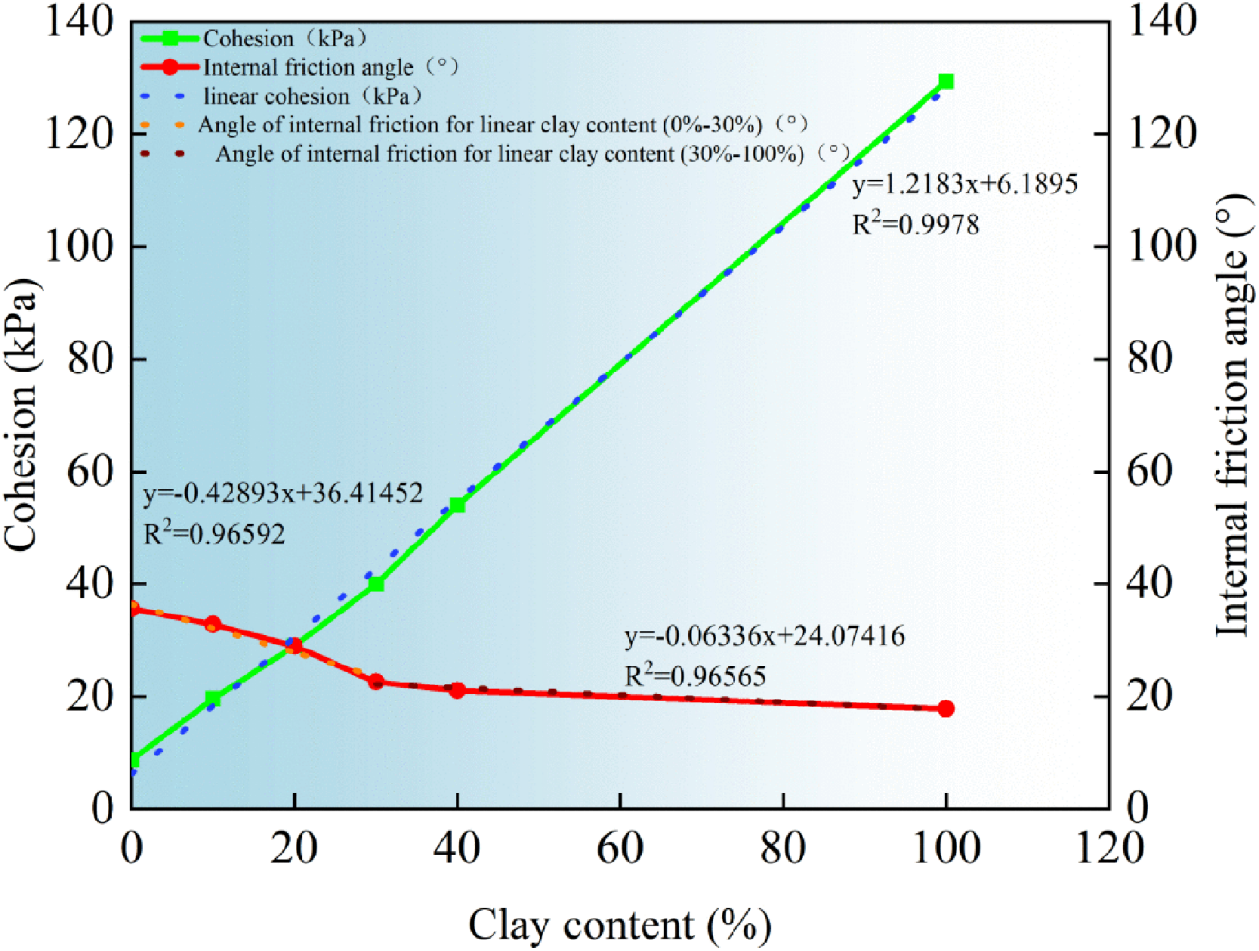
Relationship curve of clay content-cohesion force and internal friction angle.

The curves illustrate the impact of normal pressure on the magnitude of shear stress under different clay content conditions. At 100 kPa, the order of failure shear stress magnitude (corresponding to shear stress at 15% strain) is different: 100% > 0% > 10% > 40% > 30% > 20%. At 200 kPa, the sequence changes to 100% > 40% > 10% > 0% > 30% > 20%. At 300 kPa, it is 0% > 100% > 10% > 20% > 40% > 30%, and at 400 kPa, the order is 10% > 0% > 100% > 20% > 40% > 30%. This indicates that the arrangement and density of mixed soil particles are notably sensitive to normal pressure. A potential implication for practical engineering is that the differences in stress-strain characteristics at varying depths of filled soil should be given due consideration. Furthermore, cohesion and internal friction angle under different clay contents are calculated and presented in Fig 10. With increasing clay content, the cohesion of the improved soil is significantly enhanced. When the clay content increased by 40% from 0%, the cohesion rose from 8.8 kPa to 54.9 kPa, representing an increase of 523.9%. The internal friction angle gradually decreased from 35.62° to 21.54°, a reduction of approximately 39.5%. This suggests that clay content substantially impacts cohesion, possibly due to the disruption of the original structure of the granite residual soil, with cohesion being primarily affected by the fine particles. When the clay content is high, the charge suction force between them increases, leading to greater cohesion [20–21]. At the same time, the effect of the electrical double layer between soil particles will be further enhanced [22]. In fact, after adding clay to residual granite soil, its particle size distribution undergoes significant changes, which can lead to changes in shear strength [23–26]. Sun et al [27] also discovered the same phenomenon, they found that friction angle increases gradually during the process when the mineral content decreases. In fact, as the clay content increases, the mineral content also increases.

### 3.5 Microscopy results

**Fig 11:**
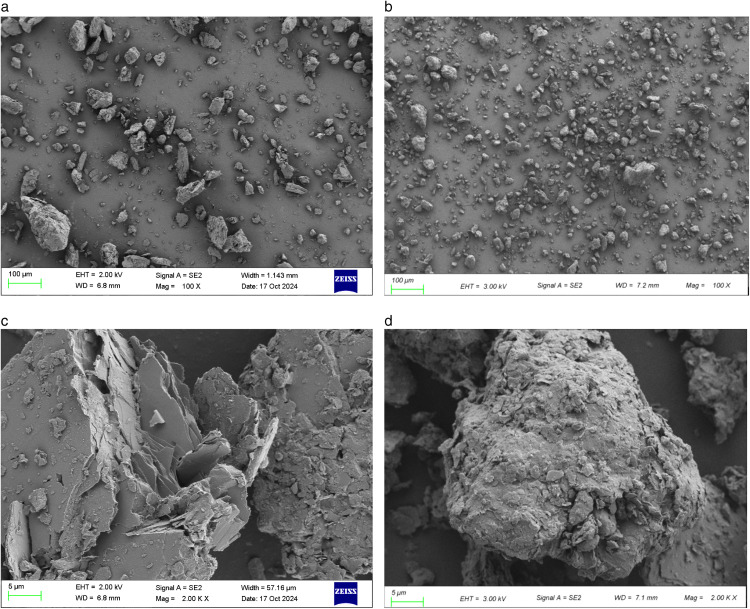
SEM images of granite residual soil and clay particles.

The granite residual soil and clay particles were observed using SEM electron microscopy scans, as shown in Fig 11. Following complete weathering, the soil reveals tiny flakes visible to the naked eye, giving the soil a relatively rough appearance. In contrast, the clay particles, composed mainly of silicates, exhibit a high degree of roundness visible to the naked eye. Microscopically, these particles are mostly circular with smooth contours and lack prominent edges or corners. From the SEM images at 100x magnification (Fig 11(a) and 11(b)), it is evident that the particle size of granite residual soil is relatively larger, with fewer cohesive particles. This observation is largely consistent with the results obtained from particle sieving analysis. Meanwhile, from the SEM image at a magnification of 2000 times (Fig 11(c) and 11(d)), the residual soil particles of granite exhibit more pronounced edges, while the surface of clay particles is relatively smooth. It should be pointed out that this microscopic analysis examines samples in a dry state. The mechanical properties of the mixture of granite residual soil and clay in a water-content state have not been discussed in this paper, which represents one of the limitations of this study. Future work will focus on investigating this aspect in greater detail.

**Fig 12:**
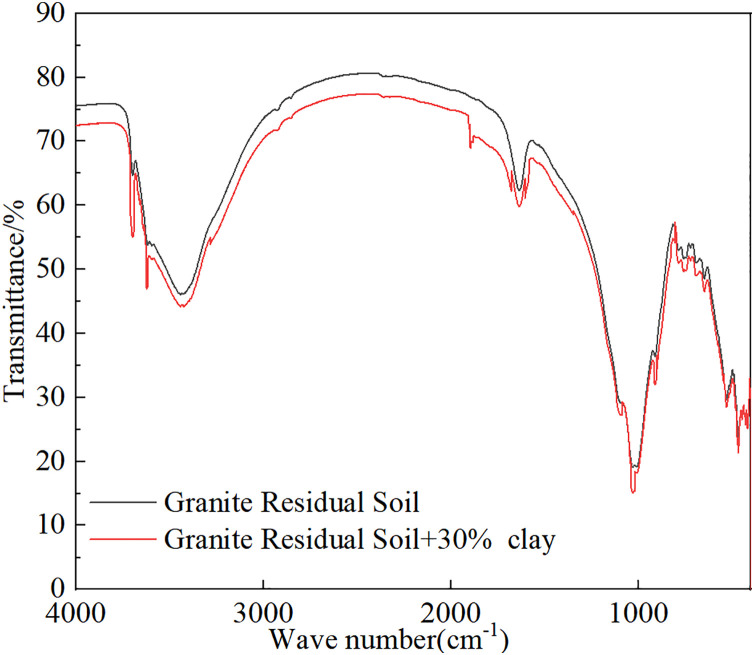
FTIR of granite residual soil with different clay contents.

The Fourier transform infrared spectroscopy (FTIR) spectra of the granite residual soil and the granite residual soil samples with 30% clay addition are presented in Fig 12. Overall, both spectra exhibit a strong and broad absorption band near 1000 cm^-1^ and a broad, gradual absorption peak in the range of 3600−3200 cm^-1^, confirming that both materials are primarily composed of silicate minerals such as quartz. The difference is that, after the addition of clay, the absorption band of the soil within the 3600−3200 cm^-1^ range becomes significantly broader and more intense, and the absorption peak near 1630 cm^-1^ is also enhanced. These changes indicate that the incorporation of clay substantially increases the content of hydroxyl functional groups and adsorbed water in the soil. The FTIR analysis results reveal that the microscopic mechanism by which clay improves granite residual soil lies in the effective enhancement of the soil’s water-holding capacity through the introduction of hydroxyl groups and adsorptive clay minerals, while preserving the original mineral composition of the residual soil.It should be noted that this test only explains the reason for the increase in soil plasticity index after improvement, and cannot explain the compression and mechanical properties.

## 4 Discussion

In this study, clay from the Qinba Mountains was selected as an improver, and the mechanical properties such as plasticity index, compactiability, compressive characteristics and shear strength of granite residual soil at different dosages were systematically explored. Unlike the common methods in existing studies that use cement, fibers or microorganisms to improve the residual soil of granite (detailed analysis is shown in Table 2), the innovation of this study lies in that it is the first to systematically explore the influence of low-cost and easily accessible natural clay on the mechanical properties of the residual soil of granite, proposes a green improvement scheme of “using local materials”, and provides detailed experimental basis for this scheme. By combining macroscopic mechanical tests with microscopic structure observations, the influence laws of clay dosage on parameters such as the liquid plasticity limit, plasticity index, compsibility, compressibility, cohesion and internal friction angle of the improved soil were systematically revealed, and the optimal dosage was determined. Microstructural analyses further reveal that the clay enhances the soil’s engineering performance through mechanisms such as particle filling and encapsulation of the granite residual soil, lubrication effects, and the promotion of hydroxyl group enrichment and adsorbed water retention at the microscale. However, this study also exhibits evident limitations. Firstly, the optimal clay ratio of 30% was determined under experimental conditions without accounting for the influence of moisture content (e.g., wet-dry cycles and climate change) on shear and compression characteristics. Secondly, due to experimental requirements, soil particles were screened to a size of 2 mm, which deviates from actual engineering conditions. In future work, we will concentrate on investigating the shear and compression behaviors of mixed soils under varying moisture contents and further conduct experimental and applied research relevant to engineering practices.

**Table 2:** Comparison of research results of the same type.

References	Materials	Cost	Key conclusions and results	The progress of this research
[28–31]	Cement	High	The cementitious substances generated by the hydration reaction of cement can effectively fill the pores of soil, enhance its strength and rigidity, and at the same time reduce its permeability.	By using local natural clay instead of industrial cementitious materials, this method can significantly reduce carbon emissions in cement production.
[32–34]	Microbial	High	The calcium carbonate precipitate generated by MICP technology can be used as a cementing material to fill the pores of soil, significantly enhancing the shear strength and water disintegration resistance of granite residual soil.	Natural clay is selected as the improvement material, and the construction process is simple, without the need for a complex biological condition control process.
[35–36]	Fiber	High	Fibers can interlock with soil particles to form a dense reinforcement network, enhancing strength through friction and mechanical interlocking. Meanwhile, the reinforcing effect of fibers can significantly enhance the impact resistance and toughness of soil. However, its high cost and strict construction requirements have limited its large-scale application in ordinary filling projects.	The materials used in this study have extremely low costs and are convenient for construction.
[37]	Clay	Low	The adsorption effect of clay can serve as a barrier material to prevent pollution.	This study focuses on enhancing the mechanical strength of granite residual soil. It aims to improve the comprehensive mechanism of soil strength through optimization of particle gradation, supplementation of cementing minerals, and enhancement of soil structure.
This study	Natural clay	Low	Clay can significantly enhance the plasticity index, maximum dry density, and cohesion of granite residual soil, while simultaneously reducing the compression coefficient of the soil mass.	A strength enhancement approach for granite residual soil was proposed, based on the principles of “local material acquisition” and cost-effectiveness. The quantitative relationship between clay content and key engineering property indicators was established. Meanwhile, the transformation process of soil physical properties from granite residual soil to clay was systematically elucidated.

## 5 Conclusions

This study investigates the residual soil derived from granite in the Qinling-Bashan Mountains of China. Through a series of systematic laboratory tests-including liquid and plastic limit, compaction, compression, and direct shear tests-combined with microstructural analyses, the mechanism of clay-based improvement of this residual soil is comprehensively examined. The following conclusions are drawn:

(1) The addition of clay can effectively enhance the water-holding capacity of granite residual soil. With the increase of clay content, the plasticity index and the optimal moisture content of granite residual soil gradually increase. This process expands the moisture content range of the soil in the formed state. Secondly, clay can effectively fill the pores of soil and enhance the compactness of the structure. With the increase of clay content, the maximum dry density of soil shows a trend of rising first and then decreasing, and reaches the maximum value of 1.87 g/cm^3^ when the dosage is 30%. The compression coefficient reaches its minimum value of 0.09 MPa^-1^ at this time.This discovery provides valuable insight for roadbed filling projects, indicating that incorporating 30% clay into local granite residual soil can optimize its compressive performance for road applications.
(2) Direct shear tests revealed that increasing clay content in the improved soil led to a linear rise in cohesion and a decline in the internal friction angle. Beyond a 30% addition ratio, the reduction in internal friction angle became less pronounced.
(3) Granite residual soil particles are mostly angular, while clay particles are more rounded. Therefore, clay particles can effectively fill the pores between soil masses and improve the particle gradation. Meanwhile, after the addition of clay, the content of hydroxyl groups and adsorbed water in the residual soil of granite increased significantly. This is the main reason for the improvement of the soil’s water-holding capacity, the increase in plasticity index and the enhancement of cohesion.

## Supporting information

S1 Data Data clay. (XLSX)
